# Impact of functional electrical stimulation on nerve-damaged muscles by quantifying fat infiltration using deep learning

**DOI:** 10.1038/s41598-024-62805-6

**Published:** 2024-05-28

**Authors:** Kassandra Walluks, Jan-Philipp Praetorius, Dirk Arnold, Marc Thilo Figge

**Affiliations:** 1 Applied Systems Biology, Leibniz Institute for Natural Product Research and Infection Biology, Hans Knöll Institute, Jena, Germany; 2 Faculty of Biological Sciences, Friedrich Schiller University Jena, Jena, Germany; 3 Institute of Zoology and Evolutionary Research, Faculty of Biological Sciences, Friedrich Schiller University Jena, Jena, Germany; 4 Clinic and Polyclinic for Otorhinolaryngology, University Hospital Jena, Jena, Germany; 5 Institute of Microbiology, Faculty of Biological Sciences, Friedrich Schiller University Jena, Jena, Germany

**Keywords:** Image processing, Machine learning

## Abstract

Quantitative imaging in life sciences has evolved into a powerful approach combining advanced microscopy acquisition and automated analysis of image data. The focus of the present study is on the imaging-based evaluation of the posterior cricoarytenoid muscle (PCA) influenced by long-term functional electrical stimulation (FES), which may assist the inspiration of patients with bilateral vocal fold paresis. To this end, muscle cross-sections of the PCA of sheep were examined by quantitative image analysis. Previous investigations of the muscle fibers and the collagen amount have not revealed signs of atrophy and fibrosis due to FES by a laryngeal pacemaker. It was therefore hypothesized that regardless of the stimulation parameters the fat in the muscle cross-sections would not be significantly altered. We here extending our previous investigations using quantitative imaging of intramuscular fat in cross-sections. In order to perform this analysis both reliably and faster than a qualitative evaluation and time-consuming manual annotation, the selection of the automated method was of crucial importance. To this end, our recently established deep neural network IMFSegNet, which provides more accurate results compared to standard machine learning approaches, was applied to more than 300 H&E stained muscle cross-sections from 22 sheep. It was found that there were no significant differences in the amount of intramuscular fat between the PCA with and without long-term FES, nor were any significant differences found between the low and high duty cycle stimulated groups. This study on a human-like animal model not only confirms the hypothesis that FES with the selected parameters has no negative impact on the PCA, but also demonstrates that objective and automated deep learning-based quantitative imaging is a powerful tool for such a challenging analysis.

## Introduction

Since the first microscopic observation of microorganisms in the seventeenth century^1^ new imaging techniques have been and are still being developed to open our eyes for the microworld. Today, we are able to observe biological processes with unprecedented accuracy allowing us to study biological and medical systems in great detail. Scientific investigations—often initiated by individual observations originating out of pure curiosity—eventually have to go beyond the level of descriptive views by rigorously quantifying the observed phenomena^2,3^. Adding the quantitative dimension to otherwise merely descriptive microscopy images enable unraveling intricate dynamic processes at the molecule-, cell- and tissue-level, and gaining insight into various functions of biomedical systems including the quantitative assessment of adverse side effects associated with medical interventions.

In this study, quantitative imaging is performed to evaluate histological samples of the sheep posterior cricoarytenoid muscles (PCA) after long-term stimulation by a laryngeal pacemaker (LP) used to assist the inspiration of patients who are affected by bilateral vocal fold paresis (BVFP). In patients with BVFP the recurrent laryngeal nerves (RLN) are transected or damaged and the PCA are paralyzed. The function of these muscles is to open the vocal folds by contracting during inspiration^4–9^, which is necessary because otherwise they remain in a median position and passively close the trachea during inspiration. A new treatment to make life more comfortable for those patients is the use of a LP, which can open the vocal folds by direct functional electrical stimulation (FES) of the PCA via reinnervating axons^4,10–12^. This allows patients to breathe in, which substantially increases their physical constitution and quality of life. A previous quantitative analysis based on fluorescence microscopy data from 22 sheep revealed that a fiber type change towards a slow-twitch muscle occurs during six months 30 Hz burst FES, but no evidence of muscular atrophy or fibrosis was detected independent of the duty cycle (DC)^13^. Nevertheless, there is a possibility that such events are masked by the replacement of atrophic muscle fibers and collagen by fat^14–18^. Although FES has been discussed for muscle recovery^19,20^ and physical activity may prevent fat infiltration^21^, it remains unclear whether this leads to intramuscular fat accumulations, which may restrict the PCA from generating sufficient tension to open the vocal folds even by using FES^17,22^. Therefore, the studies performed so far, will be extended by analyzing muscle cross-sections from our earlier study with regard to fat infiltration using a quantitative imaging approach. In the past, various qualitative and quantitative fat analyses based on Harris hematoxylin & eosin (H&E) stained cross-sections were performed^23–27^. Unfortunately, the applied analysis methods are prone to error, either due to unavoidable subjectivity of the physicians or because of the risk of misclassifying fat-free areas as fat by automated gray-scale analysis^25^. As for determining the fiber type composition a fast, automated and therefore objective quantitative imaging is also the method of choice for quantifying the intramuscular fat amount on muscle cross-sections. Due to the required analysis time, the large volume of data in typical scientific studies makes a purely manual analysis infeasible. Additionally, the subjectivity of the investigators reduces the comparability of their results, even if they would be performed with the highest possible accuracy. In contrast, a robust and automated analysis allows for the precise, objective, and expedient quantification of large-scale data sets. To ensure such accuracy, the validation of a method through the utilization of a gold standard in the form of “annotation by human experts” is a common requirement. While previous automated analyses of histological cross-sections were based on the evaluation of gray-scale intensities^25^ or color intensities using k-means clustering^28^, we recently developed an artificial intelligence (AI) based approach. In particular, building on the convolutional encoder-decoder network SegNet^29^, we trained the specialized network IMFSegNet that, even in comparison to other AI approaches, proved quantifying the spatial distribution of fat in standard H&E stained muscle cross-sections most accurately^30^. To ensure the accuracy of the results, best-practice cross-validation (leave-two-out) was performed on 100% of the data. This was done to ensure the transferability of IMFSegNet to similar data. The data used in the present study were of the exact same type (muscle sections), were generated using the same experimental protocol, and by the same human experimenters. To ensure the highest possible quality of the gold standard in the previous, a special fat stain “OilRed O” was employed to support this process. For this study we will (i) contrast the processing times for automated quantification with those obtained by a qualitative grading and by manual annotation. Additionally, we (ii) compare the consequences of k-means clustering and IMFSegNet in terms of statistical power. Then we (iii) apply the best method to perform quantitative image analysis on 326 PCA cross-sections of 22 sheep from different treatment groups to assess a possible increase in fat amount due to long-term FES with LP.

## Results

In this study, we present the results of the automated analysis of 326 H&E stained muscle cross-sections regarding the amount of intramuscular fat by comparing the PCA with (cdPCA) and without (nPCA) a cryo-damaged RLN. In addition, an LP system was implanted in sheep which was used to stimulate the muscles with different parameters. Manual and automated analyses are compared with regard to the processing times, and the accuracy of the automated quantification is evaluated for IMFSegNet in comparison with a k-means clustering approach. Finally, the fat fraction is computed to conclude the impact of RLN-damage, electrode implantation and stimulation on the PCA.

### Comparison of processing times reveals superiority of automated quantification

The processing times required to determine the amount of fat in H&E stained PCA cross-sections were estimated from a literature search^31–42^ for qualitative grading and manual annotation, and runtimes were measured for the automated approaches k-means clustering analysis and IMFSegNet. Results are compared in Table 1. The processing time for qualitative grading (see Table 1[a]) depends on the individual pathologist, but was estimated to take up to five minutes per image similar as the automated analyses by k-means clustering (see Table 1[c]) or IMFSegNet (see Table 1[d]). As expected, the two automated analyses required similar processing times and did both outcompete the processing time required for manual annotation by at least a factor 96. The processing time for the 326 images would be up to 1.3 days for qualitative grading (see Table 1[a]) and between 2 and 20 weeks for manual annotation (see Table 1[b]), without taking into account the breaking time required by a human. The time required for analyze an image result in a turnaround time for manual annotation (> 7 days) that is more than three times slower than that of k-means and IMFSegNet (< 2 days). The time required for k-means clustering is primarily caused less by the parameter estimation than by the processing of the large images for the actual parameter estimation. For images in the 10^4^ × 10^4^ pixel range, the processing time for converting the image to vectors and then adjusting the parameters accounts for most of the processing time.

**Table 1:** Processing time for muscle cross-section analysis.

Method	Processing time per image (minutes)	Processing time for 326 images (days)	Turnaround time per patient (days)
[a] Qualitative grading	0.5–5	0.2–1.2	3–5
[b] Manual annotation	60–480	13–109	> 7
[c] k-means	6.5	< 1.5	< 2
[d] IMFSegNet	5	< 1.2	< 2

The time per image corresponds to the processing time for analyzing one muscle cross-section. The processing time refers to the extrapolated processing time for 326 muscle cross-sections. Note that accounting for eight workhours per day increases the processing time for qualitative grading and manual annotation by a factor three, respectively, to 0.5–3.6 working days and 42–327 working days. The turnaround time refers to the calculated time from taking a muscle biopsy to the final clinical report, with taking in to account the breaking time required by a human and it is given for one patient. For qualitative grading and manual annotation processing times may vary due to the individual time of the pathologist and, more importantly, the total amount of fat in the muscle cross-sections. The k-means clustering and IMFSegNet were performed on an average consumer workstation (Intel Core i7-8700 K, 32 GB RAM).

### Comparison of fat infiltration reveals superiority of IMFSegNet over k-means clustering

**Figure 1:**
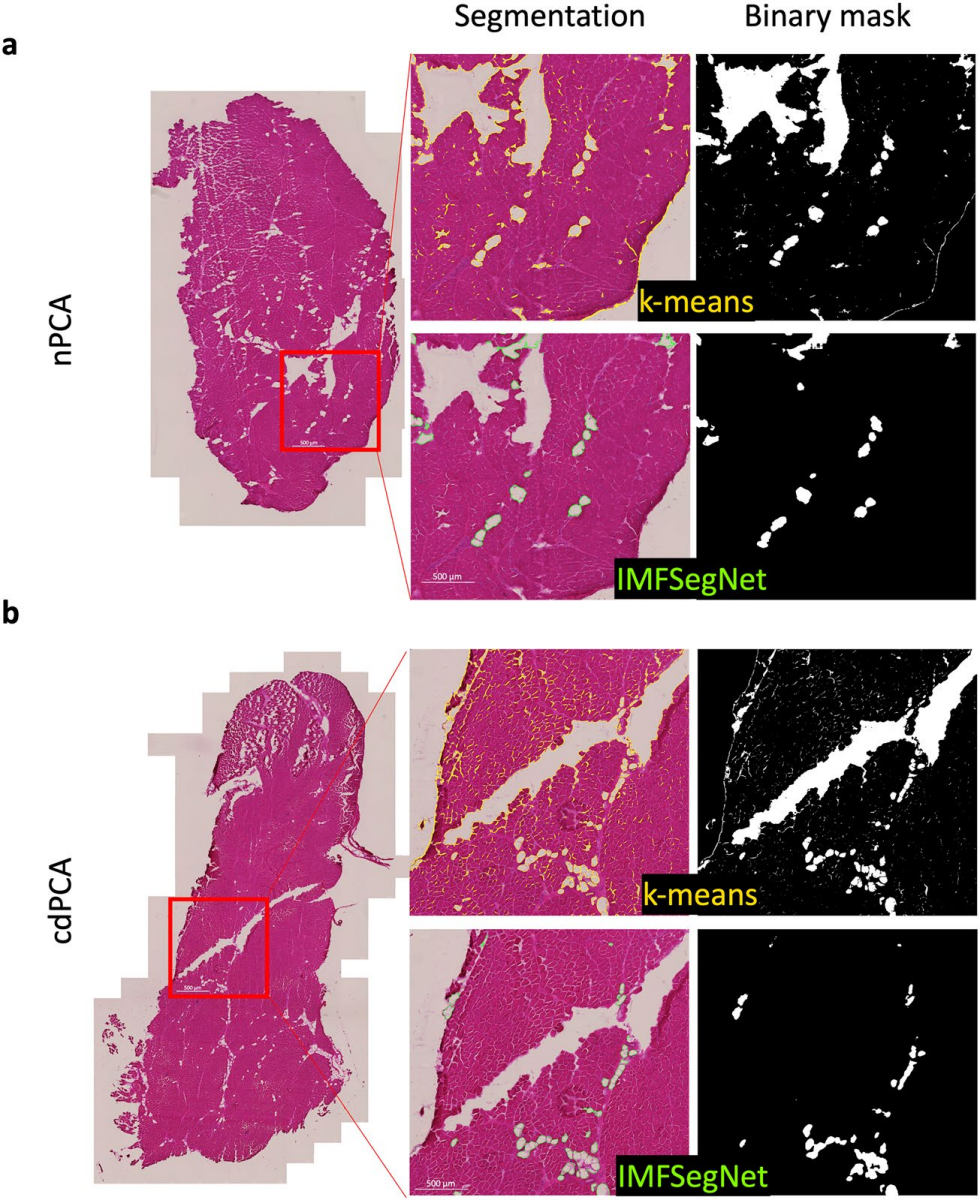
Segmented muscle cross-sections. H&E stained muscle sections with k-means and IMFSegNet predictions from control sheep (**a**) without nerve damage (nPCA) and with (**b**) nerve damage (cdPCA). The images show a representative example of segmentation determined by k-means (yellow) and IMFSegNet (green) and the corresponding binary masks.

**Figure 2:**
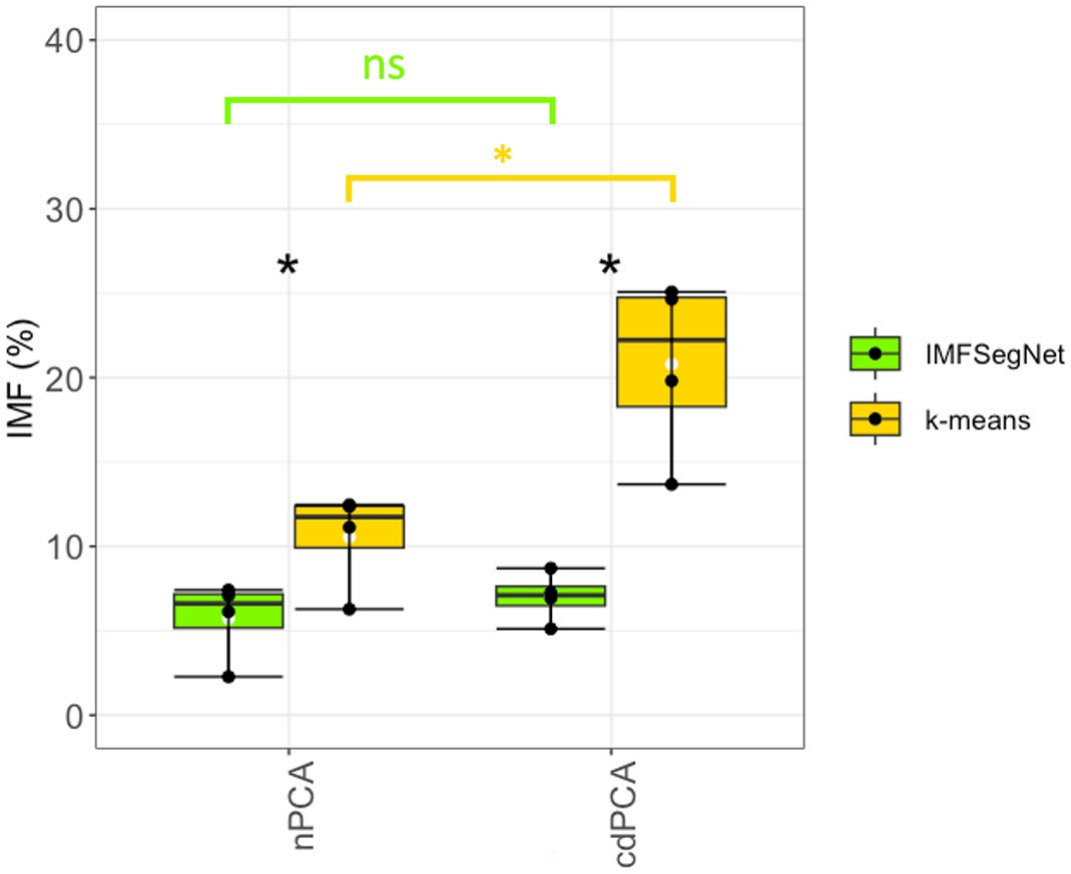
Comparison of k-means clustering and IMFSegNet. The Plot show the averaged fat amount of the nPCA and cdPCA subgroups of four control sheep (n = 4) of the PCA muscle without RLN-damage (nPCA) and with RLN-damage (cdPCA), which was determined by k-means (yellow) and IMFSegNet (green). The measured fat amount differs significantly between k-means and IMFSegNet in the nPCA (p-value = 0.04, d = 1.82) and in the cdPCA (p-value = 0.01, d = 3.54). While the IMFSegNet has no significant differences with a medium effect (p-value = 0.40, d = 0.65), k-means clustering differs significantly with a large effect (p-value = 0.02, d = 2.39). White dots denote mean values (with dot) and black lines denote the median (middle line).

The PCA samples from healthy sheep with undamaged RLN (see Fig. 1a) and cryo-damaged RLN (see Fig. 1b) were analyzed using the automated image analysis approaches k-means clustering and IMFSegNet (see Fig. 2), as described in detail in the "Materials and Methods" section. While the difference between nPCA and cdPCA in the CT group determined by k-means clustering was significant with a large effect size (p-value = 0.02, d = 2.39), no significant differences between the two groups occurred with the IMFSegNet, albeit the effect is of medium size (p-value = 0.40, d = 0.65).

The mean value of fat amount in nPCA samples as obtained from k-means clustering was 10.56% ± 2.92, which is about two times higher compared to IMFSegNet with 5.73% ± 2.37. For the cdPCA samples, the same trend was observed with the fat amount of 20.8% ± 5.32 for k-means analysis being three times higher compared to 7.01% ± 1.48 for the IMFSegNet analysis. There are significant differences with large effect sizes between the two automated analysis approaches k-means and IMFSegNet for the nPCA samples (p-value = 0.04, d = 1.82) and for the cdPCA samples (p-value = 0.01, d = 3.54). In addition, the standard deviation with the k-means analysis was by a factor 1.23 larger for nPCA samples and even by a factor 3.59 larger for cdPCA samples, indicating much more variation in the results of k-means clustering compared to the quantification by IMFSegNet. Visual inspection of fat segmentation in the muscle cross-sections (see Fig. 1) revealed that the origin of this difference is due to a clear tendency of the k-means approach to over-detection.

### Quantitative imaging by IMFSegNet reveals fat fraction

**Figure 3:**
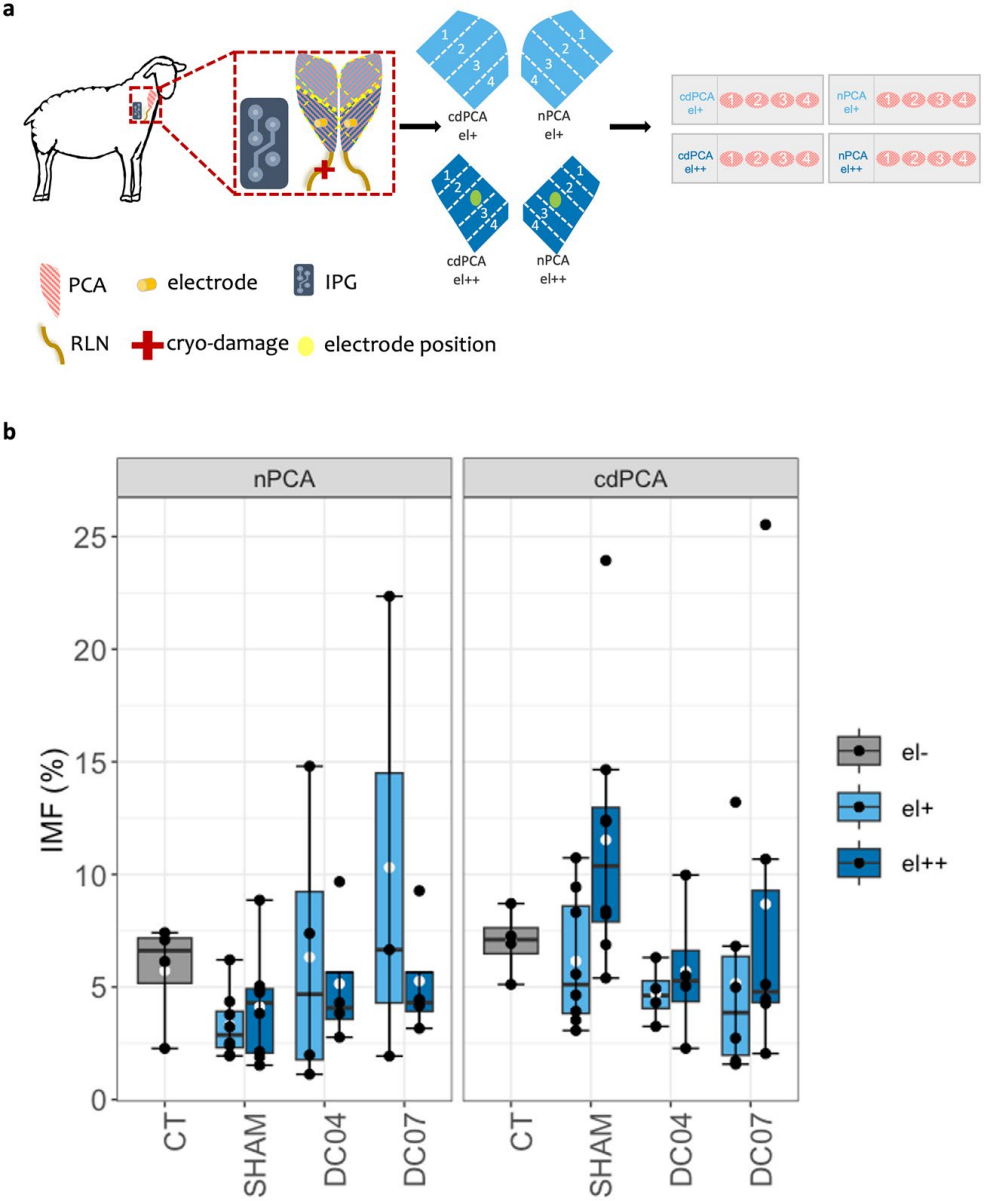
Comparison of at amount in different groups. (**a**) Graphical overview over the samples. The left PCA and right PCA samples were distinguished in in four subgroups with respect to their nerve condition without (nPCA) and with cryo-damage (cdPCA), as well as regarding the electrode position near the electrode (el++) (dark blue) and far from the electrode (el+) (light blue). Four slides with four H&E stained PCA sections for each sheep exist, which were captured and segmented with IMFSegNet. The values of each slide were aggregated by averaging to one data point. (**b**)The Plot visualizes the fat amount of the control group (CT, n = 4), the electrode group without FES (SHAM, n = 8) and the stimulated groups (DC04, n = 4 and DC07, n = 6). White dots denote mean values (with dot) and black lines denote the median (middle line).

The evaluation of the fat amount in the muscle cross-sections was determined using IMFSegNet (see Fig. 3). Differences were analyzed using the Benjamini–Hochberg^43^ correction, where the adjusted p-value is referred to as q-value^44^ with significant differences being considered for q < 0.1. We compared the fat amount between nPCA and cdPCA of all groups as well as the regions near (el++) and far the electrode (el+) for the implantation groups (see Fig. 3a).

Although the mean amount of fat in the control group (CT) (see Fig. 3b grey boxes) tended to be lower in the nPCA (mean_CT,nPCA_ = 5.7% ± 2.4) than in the cdPCA (mean_CT,cdPCA_ = 7% ± 1.5), no significant difference but a medium effect size was detected (q-value_CT_ = 0.36, d_CT_ = 0.65).

To verify whether the electrode implantation or the electrode position itself affected the amount of intramuscular fat, the PCA samples of the CT and non-stimulated implantation group (SHAM) were compared. Between the CT and SHAM group (see Fig. 3b), no significant differences were found. However, the amount of fat in the cdPCAel++ region tended to be higher than in both PCA of the CT group, which is supported by a large effect size (d_CT,nPCA/SHAM,nPCA,el++_  = 1.13 and d_CT,cdPCA/SHAM,cdPCA,el++_  = 0.90). Within the SHAM group (see Fig. 3b SHAM) the mean amount of fat was lower in the nPCA regions (nPCA: mean_SHAM,nPCA,el+_  = 3.3% and mean_SHAM,nPCA,el++_  = 4.1%) than in the cdPCA regions (cdPCA: mean_SHAM,cdPCA,el+_  = 6.2% and mean_SHAM,cdPCA,el++_  = 11.5%). The nPCAel+ region showed no significant difference and only a small effect size in comparison to the nPCAel++ region (q-value_SHAM,nPCA_ = 0.60, d_SHAM,nPCA_ = 0.41). However, due to the high fat amount in the cdPCAel++ region significant differences with large effect sizes were observed in comparison to the cdPCAel+ region (q-value_SHAM,cdPCA_ << 10^−5^, d_SHAM,cdPCA_ = 1.15) and the nPCAel++ region (q-value_SHAM,el++_  << 10^−6^, d_SHAM,el++_  = 1.64), within the SHAM group. Additionally, a significant difference with a large effect size appears between the nPCAel+ region and the cdPCAel+ region within the SHAM group (q-value_SHAM,el+_  << 10^−4^, d_SHAM,el+_  = 1.23).

To assess the impact of the stimulation intensity, we compared the influence of the stimulation with either a 12 Hz (DC04) or a 21 Hz (DC07) daily frequency equivalent on the PCA muscles. The comparison of the amount of fat in the PCA of the DC04 and DC07 group, to the CT and SHAM group revealed no significant differences (see Fig. 3b). However, the amount of fat reached a high peak value of up to 25% in the cdPCAel++ regions of the DC07 and the SHAM group. The amount of fat in the cdPCA regions of DC04 and DC07 group tended to be lower than in the SHAM and CT group, although the differences were not significant. It also appears that there is a tendency towards a difference in the nPCA region due to the increased variance in the fat amount of the two stimulation groups (DC04 and DC07) far from the electrode compared to the CT and SHAM groups, but the statistical analysis does not reveal any significant differences. Furthermore, no significant differences in the amount of fat were found between the PCA regions within the DC04 or within the DC07 group. Moreover, the comparison of the related PCA regions of the DC04 group with the DC07 group revealed no significant differences.

## Discussion

### Deep learning supported pathology segmentation

Image-based systems biology plays an increasingly important role in biological and medical research, clinical diagnosis and treatment, and continues to evolve with advances in technology and research^45^. In this study, we were able to demonstrate that quantitative imaging in the form of automated deep learning-based fat analysis is time-saving compared to conventional pathologist grading or manual analysis. Considering that automated analysis can be performed without interruption at any time of the day or night, while the pathologists analysis is limited to an eight-hour working day, this results in a further speed-up of the clinical diagnosis. While the time factor in basic research enables faster analysis, shortening diagnosis time for patients can be crucial, even though AI's much-hyped successes have not yet been widely applied in the clinic^46^.

While it is well-known that quantitative analysis is essential for modern disease grading^47^, automated and objective analyses of fat amount in muscle tissue under low-cost conditions, such as H&E staining, proved to be challenging. This challenge is primarily associated with the choice of analysis method. For example, in the present study significant differences in the amount of fat between the nPCA and cdPCA of CT group were obtained using the k-means approach, while no significant differences were predicted using IMFSegNet (see Fig. 2). When quantifying the segmented areas by k-means clustering, over-segmentation and over-detection were observed compared to visual inspection as well as statistical analysis (see Figs. 1, 2). The advantage of using the IMFSegNet instead of k-means analysis was reported before, as IMFSegNet provides the best compromise between over- and under-detection, whereas k-means is error-prone due to over-detection^30^. Using the latter approach in this case would lead to wrong conclusions regarding the long-term stimulation of the LP. Nevertheless, k-means clustering must be acknowledged for its ability to achieve comparable results, at least in the absence of artifacts such as cracks within the muscle tissue. Additionally, it does not require the use of resources for the creation of manual annotations or the training of a highly parameterized neural network. One limitation of k-means clustering is that this approach only considers the image intensities, while the IMFSegNet also utilizes structural and textural information due to its intrinsic properties as a CNN. It is important to note that the IMFSegNet undergoes an additional processing step due to its training for fat tissue recognition, a factor that suggests that it may achieve a higher level of segmentation accuracy compared to k-means clustering. The application of deep learning proved successful in many biomedical fields^48^ such as the identification and quantification of osteoclasts ^49^ and view multiple images simultaneously at different magnifications to aid in the identification and diagnosis of solid tumors^50^. In addition, open-source software tools like QuPath have gained popularity in analyzing histopathological cross-sections with deep learning techniques^51^. These achievements, along with the comparison between IMFSegNet^30^ and unsupervised k-means clustering, have made deep learning the best choice to accurately, objectively, fast and automatically analyze the amount of fat in H&E stained muscle cross-sections on previously unseen data. Therefore, this study provides clear evidence that AI-based quantitative analysis on a pre-trained network leads to more precise results in a comparatively short time. This also reduces the turnaround time, meaning the time it takes to generate the final clinical report from a muscle biopsy, which is one of the practical advantages within digital pathology imaging systems^52^.

### Functional electrical stimulation does not increase the amount of fat infiltration

To evaluate the impact of FES on the PCA the provided muscle cross-sections were divided into to the CT, the SHAM, and two DC groups as previously described (see Table 2)^13^. In all groups, the left RLN was damaged by axonotmesis. This type of nerve damage results in preservation of the nerve sheaths and reinnervation by axons from the same motoneuron pool^53^. As a result, reinnervation occurs relatively quick, after only a few weeks. Although there is no significant difference in the amount of fat between the PCAs of the CT groups, the cdPCA group still has a higher fat amount across all whiskers and quartiles. This finding is consistent with previous research indicating that nerve damage can result in atrophy, which can cause fat infiltration^18,22,54^.

**Table 2:** Sample labeling and animal numbers.

Sample group	Number of animals	Electrodes	FES	Daily frequency equivalent
CT	4	−	−	–
SHAM	8	+	−	–
DC04	4	+	+	12 Hz
DC07	6	+	+	21 Hz

*CT* control group, *SHAM* electrode group, *DC* duty cycle group, *FES* functional electrical stimulation.

The influence of the electrode implantation and the electrode position on the intramuscular fat amount was investigated by using the results of the SHAM group. The PCA muscles exhibited a comparable pattern to that of the CT group, with increased fat observed in the cdPCA compared to the nPCA throughout the distribution. Furthermore, the significant difference in this increase can be attributed to a combination of nerve damage and electrode implantation. This can be explained by the removal of damaged muscle fibers by temporarily inflammatory reactions^16^, because further studies showed a replacement of inflamed, atrophic and necrotic muscle tissue by fat^18,22,55^. However, the highest mean fat amount in the PCA of these sheep, 11.5%, did not noticeably restrict the muscles in their function, as it was shown by video-endoscopic analysis.

Finally, we wanted to know how the different stimulation parameters (12 Hz and 21 Hz) influenced the intramuscular fat amount. For this reason, the results of the DC groups were compared with those of the CT and SHAM group. Contrary to the trend observed in the CT and SHAM groups, where the cdPCA muscles exhibit more fat amount than nPCA muscles, this trend is not evident in the DC04 and DC04 stimulation groups. This leads to the conclusion that, similar to the effect on fiber type ratio^13^, electrical stimulation therefore also influences the amount of intramuscular fat.

Although, the nPCAel+ region of the DC07 group do not differ significantly from the nPCA of all other groups the variability is relatively high. The higher variability in nPCAel+ regions of DC04 and DC07 have to be addressed to the stimulation, because the RLN of that PCA were not damaged and no electrodes were implanted in that area. However, it is noteworthy that the amount of fat in all DC groups are not significantly different compared to the CT and SHAM group. Based on this evaluation of the nPCA, no obvious negative effects of long-term stimulation can be demonstrated using our stimulation parameters (12 Hz/21 Hz).

The cdPCA of both DC groups tends to contain a lower amount of fat compared to the cdPCA of the SHAM group. This indicates a beneficial effect of electrical stimulation that appears to reduce the fat infiltration induced by axonotmesis and electrode implantation observed in the cdPCA of the SHAM group. As previous studies have demonstrated a decrease in fat amount in permanently denervated and stimulated muscles^56–62^, there have also been studies that have failed to demonstrate this^15^. In this study the long-term FES appears to be large enough to reduce the amount of fat in the reinnervated PCA of DC04 and DC07 to normal levels, as a curative effect. Accordingly, the applied stimulation did not have a stronger effect on the PCA than RLN damage or electrode implantation. Also, the different daily frequency equivalents (12 Hz and 21 Hz) did not result in a discernible difference between the two DC groups.

Based on the results of the recent study revealing that the long-term FES does not lead to atrophy or fibrosis^13^, and the result of this study on a human-like animal model showing that the amount of intramuscular fat is within the normal range in both stimulated groups, i.e. in the nPCA as well as in the cdPCA, we conclude that high duty cycles can be safely used for further studies in BVFP patients.

## Conclusions

This quantitative bioimaging study is another important step in the laryngeal pacemaker development for use in humans to increase their quality of life. In addition to previous findings^13^, the analyses performed here provide further evidence that long-term use of a laryngeal pacemaker in patients with BVFP, is not to expected to result in adverse muscle events. Based on the results of the previous^13^ and this study, the clinical registration studies in human patients will now be performed with DC up to 70%. At the same time, it also provides a strong argument to use AI-based quantitative image analysis of extensive data sets with high sample size, especially for hard-to-detect objects to relieve physicians and to provide new biomedical information. Using the IMFSegNet in combination with the visual programming language JIPipe^63^, the fat amount in 326 muscle cross-sections was quantified an order of magnitude faster than manual analysis (see Table 1) and more accurate than the alternative automated analysis based on k-means clustering^30^. Another advantage of using IMFSegNet over qualitative grading is that this model, which has been specifically pre-trained for fat detection in H&E sections and verified in a best practice validation, always delivers the same output for the same input image. In contrast to a subjective grading, the same quantitative results can be delivered at any time and thus the same statistical results and conclusions can be achieved.

## Materials and methods

### Experimental data basis

This work is based on experimental data that has been generated in the context of our preclinical study on muscle atrophy in sheep after using electrical stimulation with an laryngeal pacemaker for the treatment of vocal fold paresis^13^. These data were generated in accordance with the European and German animal welfare regulations approved by the committee for animal research of the state of Thuringia, Germany (animal experimental code: UKJ-17-051), in compliance with the ARRIVE guidelines. The number of sheep was determined in accordance with the principles of the 3R framework encouraging the replacement, reduction, and refinement in animal research. The RLN of the animals were successfully cryo-damaged on one side and electrodes were implanted into the sheep PCA. For the present study, 336 histological cross-sections of PCA tissue originating from 22 non-pregnant, female Merino sheep between four and five years with a weight between 70 and 100 kg were taken. This sample pool consists of muscle cross-sections from RLN-damaged PCA (cdPCA) and PCA with natural RLN (nPCA). All provided cross-sections were divided into samples of four control sheep without electrode implantation (CT), of eight sheep with electrode implantation and no FES (SHAM) and 10 individuals with electrode implantation and 30 Hz burst stimulation with different duty cycles (DC). The PCA samples from the DC group are distinguished in four individuals with duty cycle of 40% (DC04; daily frequency equivalent: 12 Hz) and in six individuals with duty cycle of 70% of the respiratory cycle (DC07; daily frequency equivalent: 21 Hz,). From the groups with electrode implantation, there exist samples near the electrode (el++) and far from the electrode (el+) (see Table 2 and see Fig. 3a). In the DC07 group 2 slides of the nPCA were missing for two animals.

Before staining, the frozen cross-sections were thawed for 30 min and fixed with 10% formalin for 5 min. The cross-sections were stained with H&E staining^64^. The slides are covered with coverslips (strength 1, 24 × 50 mm, Carl Roth GmbH, Germany) and DPX (non-aqueous medium for microscopy, Sigma–Aldrich). The stained cross-sections were imaged using a ZEISS AxioScan 7 (Zeiss microscopy GmbH, Jena, Germany) with a magnification of ×20 and a resolution of 0.442 × 0.442 µm per pixel. After imaging 326 out of 336 muscle-sections were analyzed. Eight images were discarded because they either contained large air bubbles or dirt, or because a section of the slide was wrinkled or destroyed. Two images were excluded for technical reasons as they could not be analyzed with a common library in Python.

### Automated fat segmentation of whole PCA muscle tissue according to FAIR principles

The segmentation of the fat in muscle tissues was performed to determine the fat fraction for each muscle cross-section. To ensure the FAIR^65^ principles with regard to the reproducibility of automated analyses, a software environment that can deal with this challenge is needed. We used the visual programming language JIPipe^63^, which provides FAIR batch processing for ImageJ^66^. JIPipe enables biomedical image analysis for non-programmers and transfers short and simple as well as comprehensive analysis pipelines to a user-friendly level by flowcharts of image analysis operations.

To avoid potential over-detection of muscle tissue, we manually annotated regions with heavy pollution or areas of cartilage in 22 samples and excluded those regions from the analysis. Initially, the original images of muscle cross-sections were converted to a common color space to obtain a binary image that can distinguish tissue from background. The tissue can subsequently be visually examined and separated using a threshold value of 15% in the saturation channel. We used remove outliers 2D from ImageJ, which is designed to correct dead pixels, in order to remove the remaining artifacts. A window size of 31 pixels was determined, which defines the surroundings of a pixel to be examined, whether it is considered noise and therefore as background. This threshold value guaranteed that the operator removes noise because it corresponds to objects that are less than 12% smaller than typical fat cells in size. For closing holes in perforated muscle tissue smaller than or equal to 5% of the average fat cell size, we applied morphological closing operation with a kernel size radius of 13 pixels. To minimize over-segmentations caused by the fat prediction, we determined the convex hull of the final tissue mask and kept only the predictions that were within this convex hull. Following this, the total area of each muscle tissue was quantified.

### Comprehensive and automated fat characterization in sheep muscles

We applied two segmentation methods in a comparative fashion. On the one hand, the fat amount was first quantified for with the color-based k-means clustering^28^. Each image was converted to CIELAB color space, to perform k-means clustering on the color intensities using the Python library “scitkit-learn”. The only required parameter of this unsupervised approach is the number of clusters, which in our case was set to three (fat, tissue, and background). On the other hand, we applied our previously established neural network IMFSegNet, which was built on the convolutional encoder-decoder network SegNet^67^. IMFSegNet was demonstrated to be generally applicable and was successful in detecting fine-grained features in a data-driven manner. Based on a best-practice cross-validation, IMFSegNet was evaluated with particular focus on reproducibility to be the method of choice for analyzing big data sets of histological tissue cross-sections.

### Statistical calculation

The amount of fat per muscle cross-section was measured as a percentage of the size of the muscle cross-section and then used for a comprehensive statistical analysis. Statistical tests were performed with R v4.1.0. For the comparison of k-means (sklearn version 1.0.2) and IMFSegNet (tensorflow-gpu version 2.5.1) a t-test was performed and Cohen’s d^68^ was calculated. Statistical significance was assumed for p < 0.05. As the analysis included multiple cross-sections per sheep and various experimental conditions the p-values for the fat analysis were calculated using a linear mixed model with the animal being a random factor. The degrees of freedom were determined with the Satterthwaite’s method^69^. The Benjamini–Hochberg[43]correction was used to define the false discovery rate and to calculate adjusted p-values (q-values)^44^. Significance was considered at q < 0.1. In addition, pairwise effect sizes were determined using Cohen’s D (d), where d indicates a small effect size at approximately d = 0.2, a medium effect size at approximately d = 0.5, and a substantial effect size at approximately d = 0.8 or greater.

## Data Availability

The datasets generated during and analysed during the current study are available in the repository https://asbdata.hki-jena.de/WalluksPraetoriusEtAl2023_ScientificReports.
